# The Partisan Impact on Local Government Dissemination of COVID-19 Information: Assessing US County Government Websites

**DOI:** 10.1017/S0008423920000918

**Published:** 2020-10-12

**Authors:** Michael A. Hansen, Isabelle Johansson, Kalie Sadowski, Joseph Blaszcynski, Sarah Meyer

**Affiliations:** 1 Politics, Philosophy, and Law Department, University of Wisconsin—Parkside, 900 Wood Road, Kenosha, WI, 53144; 2 Department of Sociology, Lund University and Department of Social Science, Kristianstad University, Högskolan Kristianstad, 291 88 Kristianstad, Sweden; 3 University of Wisconsin—Parkside 900 Wood Road, Kenosha, WI, 53144

**Keywords:** partisanship, COVID-19, county websites, local governments, partisanerie, COVID-19, sites Web de comté, gouvernements locaux

## Abstract

This study explores the relationship between local government dissemination of COVID-19 information and partisanship. The unit of analysis is all official county government websites in the United States. In particular, we investigate if there is a correlation between the overall partisanship of a county and whether a county government's website (1) mentions COVID-19 and (2) provides safety instructions concerning COVID-19. We hypothesize that mass partisanship will impact the probability that a county government's website provides information related to the coronavirus. We find that a larger share of Democratic voters in a county is associated with an increase in the probability that a county government's website mentions COVID-19 and provides safety instructions for its residents. The results hold even after controlling for population density, internet subscriptions and COVID-19 cases and deaths. The finding indicates that citizens’ access to information, even on matters of public health, are partially a consequence of partisanship.

## Resumé

Cette étude explore la relation entre la diffusion d'informations sur la COVID-19 par les gouvernements locaux et la partisanerie. L'unité d'analyse est l'ensemble des sites Web officiels des gouvernements de comté aux États-Unis. En particulier, nous cherchons à savoir s'il existe une corrélation entre la partisanerie générale d'un comté et le fait que le site Web d'un gouvernement de comté 1) mentionne la COVID-19 et 2) donne des instructions de sécurité concernant la COVID-19. Nous émettons l'hypothèse que la partisanerie de masse aura un impact sur la probabilité que le site Web d'un gouvernement de comté fournisse des informations relatives au coronavirus. Nous constatons qu'une plus grande proportion d'électeurs démocrates dans un comté est associée à une augmentation de la probabilité que le site Web d'un gouvernement de comté mentionne la COVID-19 et fournisse des instructions de sécurité pour ses habitants. Ces constatations subsistent même après contrôle de la densité de population, des abonnements à Internet et des cas et décès liés à la COVID-19. Les résultats indiquent que l'accès des citoyens à l'information, même sur les questions de santé publique, est en partie une conséquence de la partisanerie.

From the outset of the coronavirus pandemic to today, President Donald Trump's statements and actions have consistently downplayed the seriousness of the potential spread of COVID-19. For instance, in a press conference on February 26, 2020, while discussing the virus, the president stated, “And again, when you have 15 people, and the 15 within a couple of days is going to be down to close to zero, that's a pretty good job we've done” (Trump, 2020). Over the past seven months, conservative news outlets and a number of Republican politicians have adopted the president's rhetoric downplaying the virus. In fact, the president and conservative news outlets have been overt opponents of states’ actions to remain under “safer at home” orders, attacking governors and local officials that support these policies. These attacks have led scholars to investigate the relationships between partisanship and public policy choices, citizen and elite attitudes and knowledge, and citizen and elite behaviour related to COVID-19 (Armstrong and Lucus, 2020; Gadarian et al., 2020; Merkley et al., 2020; Miller, 2020; Motta et al., 2020; Pickup et al., 2020; van der Linden and Savoie, 2020). One consistent finding has been that the mass public's attitudes have become increasingly polarized on the issue of COVID-19 (Gadarian et al., 2020; Miller, 2020; Motta et al., 2020; van der Linden and Savoie, 2020).

To further our understanding of the link between partisanship and issues related to COVID-19, we explore the relationship between mass partisanship and local government dissemination of COVID-19 information on official county government websites in the United States. In particular, we investigate if there is a correlation between the overall partisanship of a county and whether a county government's website (1) mentions COVID-19 and (2) provides safety instructions concerning COVID-19. We hypothesize that a county population's partisanship will impact the probability that a county government's website provides information related to COVID-19. We find that a larger 2016 Hillary Clinton vote share for a county is associated with an increase in the probability that a county government's website will mention COVID-19 and provide safety instructions for county residents, even after we control for population density, internet subscriptions and COVID-19 cases and deaths.

## Local Government and Mass Partisanship

Research provides evidence that the behaviour of local-level elected representatives is related to the partisanship of the local-level population. Einstein and Kogan (2015) investigate more than 2,000 midsize cities in the United States and find that cities with a more Democratic population have significantly higher spending across a number of service areas. Similarly, Tausanovitch and Warshaw (2014) find that policy adoption in cities and towns with a population over 20,000 is predicted by the partisanship of the population on a liberal–conservative dimension. The findings hold across different institutional arrangements at the local level. The authors also show that presidential vote share is strongly related to the ideological profile of the city on the liberal–conservative dimension (Tausanovitch and Warshaw, 2014).

For counties, a similar relationship between the partisanship of the population and actions of elected officials is clear in current research. Percival et al. (2009) explore counties in California. The authors find that social service spending is a function of ideological orientation and that liberal counties are more likely to increase spending on welfare and social services. Choi et al. (2010) confirm these findings. Their investigation of Florida counties finds that presidential and gubernatorial vote share for Democratic candidates has a positive impact on local-level spending. Finally, Ybarra and Krebs (2010) test for a relationship between partisanship and policy that goes beyond fiscal policy. They find that Democratic vote share is predictive of a county being more likely to ban smoking in non-hospitality workplaces. The results of these studies indicate that a local-level population's partisanship may have an impact on elected officials’ behaviour on a range of actions.

## County Government Websites

Why explore local government websites? Local governments have the power to close, alter the hours of operation and provide additional restrictions and rules for both public and private entities to deal with COVID-19. In fact, following the Wisconsin Supreme Court's ruling on Governor Tony Evers’ “Safer at Home” order, local governments are the only entity left in the state of Wisconsin with the power to enforce such COVID-19 related policies (Wisconsin Supreme Court, *Wisconsin Legislature v. Palm*, 2020). One could imagine that legal challenges to governors’ policies in other states could end in a similar outcome. Since citizens have interactions with local government offices, public parks and buildings and/or private businesses and entities on a near daily basis, citizens need to be aware of local government policies in order to follow the law. County government websites should publish information that allows citizens to respect the laws and regulations in place. During a pandemic, one would expect information about any county policy that has been adopted as a result of the virus. Currently, information on COVID-19 cases and deaths is reported at the county level in the United States. Since county governments have an obvious incentive to reduce the spread of the disease, one would expect that county governments would make a similarly active attempt to educate their citizens on how to stay safe.

What predicts the level of information available on local government websites generally? There are only a handful of studies that investigate the level of information available on county or local government websites, and they are limited to a subset of counties in the United States (Harder and Jordan, 2013; Bernick et al., 2014; Lowatcharin and Menifield, 2015; Baker and Chin, 2016; Bearfield and Bowman, 2017). These studies mainly focus on financial transparency, county board meeting minutes and other general information that is measured at the county level, such as tax information, crime records, and so on. These studies find no link between county government resources, digital infrastructure or county-level citizen demographics (including age, race, education level, household income and poverty) and greater levels of information (Harder and Jordan, 2013; Baker and Chin, 2016). However, one consistent finding is that population or population density is associated with greater levels of information on government websites (Lowatcharin and Menifield, 2015; Baker and Chin, 2016; Bearfield and Bowman, 2017). In addition, Bernick et al. (2014) find that the mass partisanship of a county, measured as Obama's 2012 vote share, is associated with greater fiscal transparency on county websites.

## Partisanship and COVID-19

There are a number of studies that demonstrate the impact that elite cues have on attitudes in the United States (Zaller, 1992; Mondak, 1993; Berinsky, 2009). These studies indicate that political elites have considerable power to frame an issue, influence public opinion in a particular direction and induce behaviour among citizens (Zaller, 1992; Mondak, 1993; Berinsky, 2009; Hansen and Dolan, 2020). As stated previously, the messaging delivered by President Trump and other prominent Republicans has consistently downplayed the seriousness of COVID-19. In fact, Green et al. (2020) find party polarization in the cues sent out by members of the US House of Representatives and Senate on the issue of COVID-19 on Twitter. In particular, the authors find that Democrats are more likely to mention threats to public health, individualized safety and American workers. Republicans are more likely to mention China, businesses, blame and opening the economy. The authors are able to correctly predict partisanship 76 per cent of the time based on the content of a tweet on COVID-19, which indicates that the response to COVID-19 aligns with the continued trend toward partisanship regarding societal issues (Green et al., 2020). Notably, the partisan impact on attitudes toward COVID-19 appears to be specific to the United States when compared to Canada. Recent studies by Armstrong and Lucas (2020), Merkley et al. (2020) and Pickup et al. (2020) demonstrate that there is either no or little to no partisan divide on the issue of COVID-19 in Canada in comparison to the United States.

There have been several studies that demonstrate that the polarization of elites on the issue of COVID-19 extends to the media and the public. Motta et al. (2020) demonstrate that right-wing media, such as Fox News and Breitbart, regularly discussed misinformation about COVID-19 in the early days of the pandemic. The authors’ study finds that people who consumed right-wing media sources in the early days of the pandemic were more likely to endorse COVID-19 misinformation and to think that the health risks were being exaggerated.

These findings extend to additional studies that confirm a partisan divide in society in regard to COVID-19. A study from the Pew Research Center finds that 63 per cent of Republicans believe that the outbreak has been exaggerated, compared to just 18 per cent of Democrats who do (Mitchell et al., 2020). Further, the study finds that 68 per cent of people who indicate that they receive their information on COVID-19 from Trump believe that the outbreak has been made into a bigger deal than what is actually the case, compared to 33 per cent of people who receive their news elsewhere (Mitchell et al., 2020). Similarly, Miller (2020) finds that Republicans are more likely to believe in COVID-19 conspiracy theories due to their stronger political motivation to endorse such theories.

The partisan gap between Democrats and Republicans extends from attitudes to a range of behaviours. Gadarian et al. (2020) find that partisanship is the single most consistent factor that differentiates Americans’ policy preferences and behaviours related to COVID-19. The study reveals that Democrats are more likely to indicate increases in activities such as washing hands, avoiding contact with others, avoiding gatherings, information seeking and self-quarantining (Gadarian et al., 2020). Finally, van der Linden and Savoie (2020) find that partisanship is a more substantive predictor of mask usage among individuals than is collective interest or self-interest. Their study finds that Democrats are more likely to report wearing a mask to prevent the spread of the virus. Given these results in previous studies, we expect that counties that are more Democratic will be more likely to provide information on COVID-19 on their websites.

## Dependent Variables and Method

We explore two dependent variables. First, we code every county government website in the United States for whether the website mentions COVID-19.^1^ The variable was coded as 1 if there is any mention of COVID-19 on the website and as 0 if there is no mention of COVID-19. We explore all website pages that are connected to the main page. We generously code the mentioning of COVID-19. For example, if there is a statement indicating that a building is closed due to COVID-19, such as the circuit court being closed on the law enforcement webpage, we code that variable as 1. In addition, we make use of the search function on these websites and search for use of the word *COVID* in order to verify whether it was mentioned anywhere on the website.

Second, we code each website for whether we could find information on how to stay safe during the spread of the virus. The variable is coded as 1 if there is any information on staying safe from COVID-19 and as 0 if there is no such information. Again, we explore all website pages that are connected to the main page. In addition, if any of the county website pages contain an external link to guidelines on staying safe from the virus, we code the variable as 1. This occurrence was rare at the time period that we were coding the variable. However, there were a few instances where a health services webpage on the county website provided a link to safety precautions on the state's health department webpage. Finally, we make use of the search function to verify whether safety guidelines were provided anywhere on the website. The variables were coded between April 17, 2020, and April 24, 2020. The variables were coded during this condensed time period so that we could be sure that any variation in the availability of information was not a consequence of timing. We estimate individual multiple logistic regression models to predict the dependent variables.^2^

For each of the two variables, three researchers coded one-third of the overall county websites (around 1,000 cases). At the same time, a fourth researcher was assigned to code 600 county websites, with 200 counties randomly assigned from each of the three samples representing one-third of the overall data. The fourth researcher's sample was utilized to provide a coder reliability test. The intercoder reliability test uncovered zero disagreements between the coders. The simplicity of the county websites and the researchers’ use of website search functions eliminated coding errors.

## Independent Variables

There are five independent variables included in the multiple regression analysis. First, since previous studies exploring county government website information find that population density has an impact on the level of information (Choi et al., 2010; Lowatcharin and Menifield, 2015; Baker and Chin, 2016; Bearfield and Bowman, 2017), we include it in the analysis. The expectation is that a larger population density is associated with providing information. Next, we include two measures of COVID-19 prevalence in a county at the time of exploring the website: (1) COVID-19 cases and (2) COVID-19 deaths (Johns Hopkins University, 2020).^3^ Since the two variables are correlated higher than 0.80, we estimate separate models with each of the two variables. In addition, the variables are log transformed to address issues of non-normality.^4^ The prediction is that a higher number of cases or deaths resulting from the virus will be associated with greater information about the virus on the county government's website. Our fourth independent variable is the proportion of each county's residents with broadband internet subscriptions (US Census Bureau, 2018). The expectation is that counties with a larger proportion of residents who have an internet subscription will be more likely to utilize their official websites to disseminate information to their residents.

Finally, a measure of mass partisanship is included in the analysis. In line with previous studies that explored partisanship's impact on county government website information (Bernick et al., 2014) and studies that demonstrated how a population's partisanship impacts local-level elected official behaviour (Choi et al., 2010; Ybarra and Krebs, 2010), we used Hillary Clinton's 2016 county vote share as a measure of mass partisanship.^5^ Clinton's county-level vote share was easily obtained from a number of sources, including states’ election commission websites. To verify that Clinton's 2016 election was not an anomaly in terms of Democratic vote share, we test the correlation between Clinton's 2016 county vote share and Barack Obama's county vote share in 2012 and 2008. We find that Clinton's 2016 vote share correlated with Obama's 2012 vote share at 0.94 and with his 2008 vote share at 0.90. The results indicate a clear partisanship pattern for the counties. Our hypothesis is that even when controlling for population density, internet subscriptions and COVID-19 cases and deaths, citizens of counties with a higher Democratic vote share will be more likely to receive information on the virus.

It is important to acknowledge that a number of studies uncover a relationship between elite partisanship at the local level and local-level government behaviour (de Benedictis-Kessner and Warshaw, 2016; Einstein and Glick, 2018; de Benedictis-Kessner and Warshaw, 2020). Therefore, for a subset of the sample, we test the correlation between Clinton's 2016 vote share and county government partisanship using a measure for the Democratic proportion of the county council variable for years 2012–2016 from the de Benedictis-Kessner and Warshaw (2020) study. We find that the two variables correlate at 0.73, which indicates high congruence between the population's partisanship and local elected official partisanship.^6^ That being said, it is important to recognize that this measure was only available for less than 10 per cent of the counties. Further, an attempt to use Clinton's 2016 vote share as a measure of elite level partisanship would be a crude proxy measure. Thus, we focus on the relationship between the population's partisanship and the availability of information on COVID-19 in the results section.

## Results

**Figure 1:**
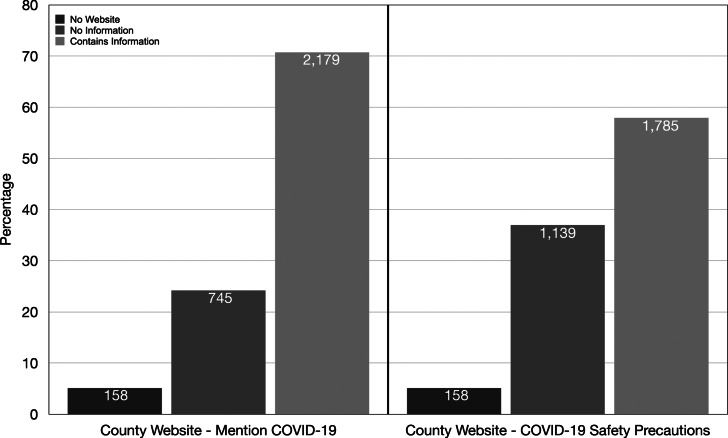
County Government Website Information on COVID-19

In all, the analysis explores 3,082 county governments (Figure 1).^7^ Out of the 3,082 county governments in the United States, only 158 (5.1 per cent) had no official website. The two variables measuring the prevalence of information on COVID-19 on county government websites contained substantial variation. When exploring the counties that had official county government websites, 25.5 per cent (745) of these websites did not mention COVID-19. Rather surprisingly, more than one-third (39 per cent, or 1,139) of county government websites did not provide any information on, or link to, safety precautions in regard to the virus.

**Figure 2:**
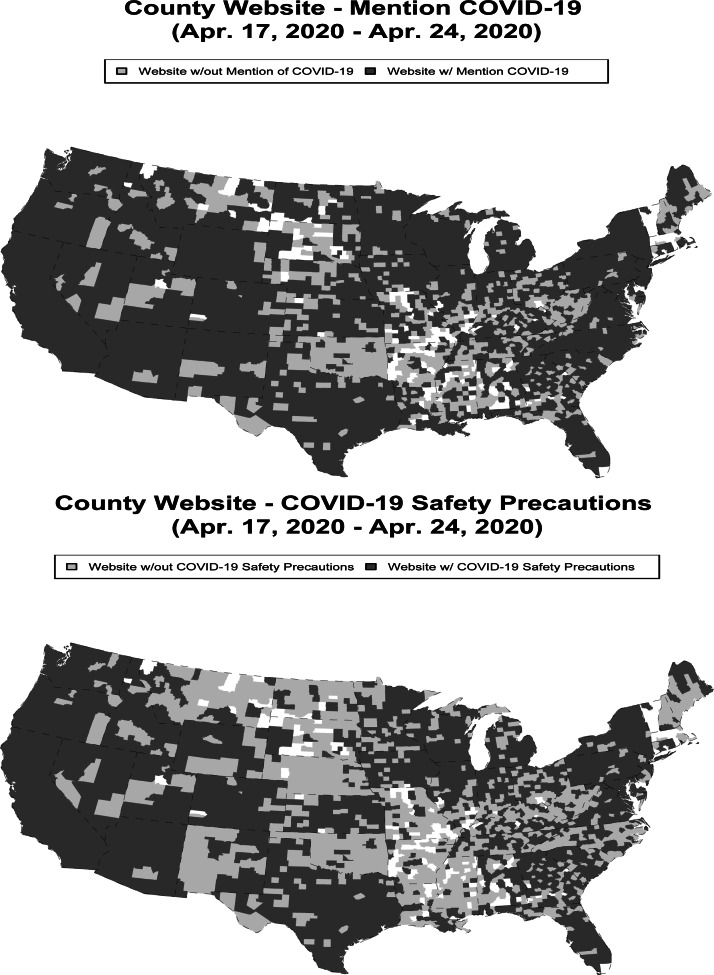
County Government Website Information on COVID-19: Map of Counties *Note:* The colour white indicates that the county did not have an official government website. Alaska only had one government website without information, and all Hawaiian government websites contained information.

In Figure 2, we plot whether counties had mentioned COVID-19 on their website or provided safety information for the US mainland. As the top of Figure 2 displays, the counties that do not provide any mention of COVID-19 on their website are concentrated quite heavily in the central to central/southern United States. However, there are counties throughout the country that do not mention COVID-19 on their websites. In the bottom of Figure 2, we see that a lack of mention of safety precautions on county government websites is geographically dispersed. In almost every state, there is at least one county that does not provide safety information on their website.

In Table 1, output is displayed from four models predicting whether county government websites mention COVID-19 or provide safety precautions related to COVID-19.^8^ The results indicate that population density has no relationship with county government websites mentioning COVID-19. Surprisingly, there is a negative relationship between population density and whether a county provides safety precautions on its website. In line with expectations, as the proportion of the households in the county that have internet subscriptions increases, the likelihood that a county's website mentions COVID-19 and provides safety precautions increases.

**Table 1:** Models Predicting County Websites Mentioning COVID-19 and Providing Safety Information

	Mention COVID-19		Provide safety precautions	
(Intercept)	−5.23*	−5.30*	−4.49*	−4.70*
	(0.43)	(0.44)	(0.37)	(0.36)
Population density	0.00	0.00	−0.00*	−0.00*
	(0.00)	(0.00)	(0.00)	(0.00)
Prop(households with internet)	7.20*	7.40*	5.58*	5.98*
	(0.57)	(0.57)	(0.50)	(0.48)
log(COVID-19 cases)	0.08*		0.12*	
	(0.03)		(0.03)	
log(COVID-19 deaths)		0.07		0.16*
		(0.05)		(0.04)
Clinton vote share	0.03*	0.03*	0.02*	0.02*
	(0.00)	(0.00)	(0.00)	(0.00)
*N*	2,924	2,924	2,924	2,924
AIC	2986.05	2989.95	3629.64	3634.10
BIC	3105.74	3109.63	3749.32	3753.79
Log likelihood	−1473.03	−1474.97	−1794.82	−1797.05

*Note:* * indicates statistical significance at *p* < .05; standard errors in parentheses.

As expected, there is a positive relationship between the amount of COVID-19 cases in a county and whether a county government website mentions COVID-19 and provides safety information. For COVID-19 deaths, the results indicate that they are not related to mentioning the virus. However, there is a positive relationship between the number of COVID-19 deaths and a county government providing safety information on their website.

**Figure 3:**
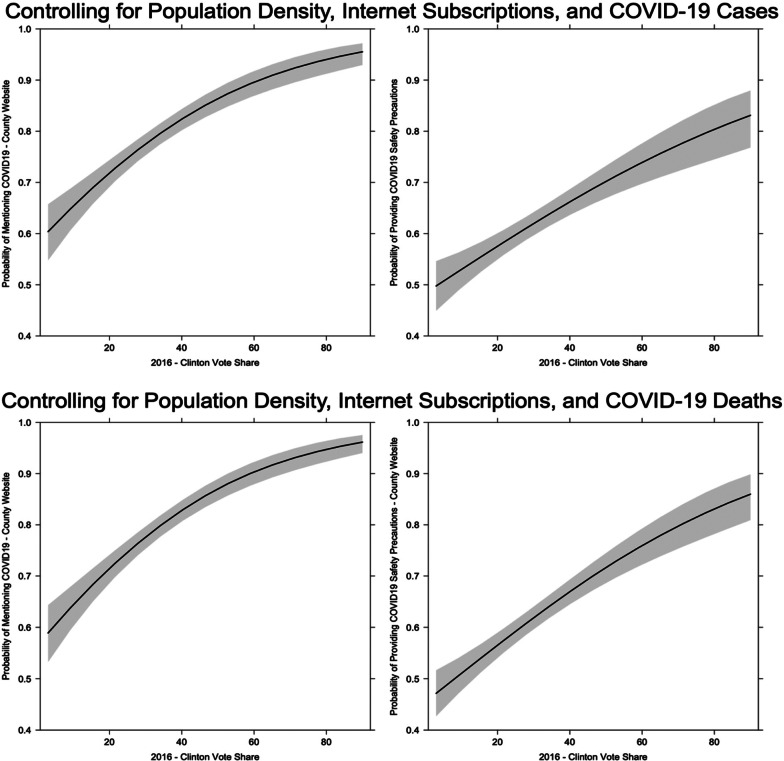
The Effect of Partisanship on County Government Website Information on COVID-19

The results from Table 1 provide evidence for our hypothesis. The results indicate that there are positive statistically significant relationships between 2016 Clinton vote share and both mentioning COVID-19 on the county government website and providing safety information related to the virus. The result holds even when controlling for COVID-19 deaths and cases. The substantive impact of the vote share variable is quite large. In Figure 3, we plot the predicted probabilities of the Clinton vote share variable for the four models presented in Table 1.^9^ First, when controlling for population density, proportion of internet subscriptions, and COVID-19 cases, Figure 3 indicates that when comparing a county where Clinton received her lowest vote share to a county where Clinton received the highest vote share, there is an increase in the probability that a county website mentions COVID-19 of around 0.3. Similarly, when moving from the lowest to the largest values of vote share for Clinton, there is an increase of 0.25 in the probability that a county's website provides safety information related to COVID-19.

In the lower segment of the graph, predicted probabilities are plotted for the vote share variable when we control for population density, proportion of internet subscriptions, and COVID-19 deaths. When exploring COVID-19 deaths rather than the number of cases, partisanship is a powerful predictor of information on county government websites. The increase in the probability that a county's website mentions COVID-19 is larger than when we control for cases at 0.40. That being said, the increase in the probability that a county website contains safety information when comparing a county with the lowest Clinton vote share to the largest Clinton vote share is similar, at around 0.3. The results indicate that there is a clear statistical relationship between the mass partisanship of a county and the information available to citizens from local governments. In particular, citizens of predominately Democratic counties are more likely to receive information on COVID-19 than are citizens of predominately Republican counties.

## Concluding Remarks

Our analysis builds upon the recent work of scholars who find relationships between partisanship and public policy choices, citizen and elite attitudes and knowledge, and citizen and elite behaviour related to COVID-19 (Armstrong and Lucas, 2020; Gadarian et al., 2020; Merkley et al., 2020; Miller, 2020; Motta et al., 2020; Pickup et al., 2020; van der Linden and Savoie, 2020). In addition, we contribute to the broader research on the relationship between a population's partisanship and local government behaviour (Percival et al., 2009; Choi et al., 2010; Ybarra and Krebs, 2010; Tausanovitch and Warshaw, 2014; Einstein and Kogan, 2016). Here, we investigate county websites throughout the United States to understand variance in local government dissemination of information relating to the current pandemic. We find a positive relationship between Democratic vote share in the 2016 election and a county government's dissemination of COVID-19 information on its website. On average, the results indicate that citizens in predominately Republican counties have less opportunity to acquire information and knowledge about the virus on official local government websites. The results demonstrate that even at the local level, partisanship plays a substantive role in the United States’ current handling of the pandemic.

## Notes

1 In the state of Alaska, county governments are referred to as *boroughs* and *census areas*, and in Louisiana they are referred to as *parishes*.

2 Fixed effects models accounting for variance by state were estimated as a robustness check. The results confirmed the findings that we present here.

3 As it was relatively early in the pandemic, the mean number of cases was 265.4 and the median number was 12. For deaths, the mean number of deaths was 13.38 and the median was 0.

4 Since there are zero values in the dataset for the COVID-19 cases and deaths variables, we take the log(cases/deaths − MinimumValue(cases/deaths) − 1).

5 We also explored the impact of demographic data at the county level on a subset of counties from the dataset, including median age, education and income level. However, we did not find any meaningful, consistent results worth including in the research note. Population was also included as an independent variable, but the results were substantively the same as population density.

6 Since only two-thirds of states have partisan elections and some of those states have a mix of partisan and nonpartisan elections, we decided to use the traditional presidential vote share measure as a measure of partisanship rather than removing over one-third of the overall sample. As the correlation tests demonstrate, this measure is highly predictive of the county's overall partisanship as well as the partisanship of county governments.

7 We include large single-tier municipalities, such as Houston, in our analysis. Further, when there was debate about including a single-tier municipality due to an issue with a Federal Information Processing Standards (FIPS) code, we double-checked that there was no difference between the municipality and the corresponding county in disseminating information.

8 Additionally, we estimated models where we coded counties that did not have a website as not mentioning COVID-19 or providing safety precautions. The results are substantively similar to the results presented here where the counties without websites are not included in the analysis.

9 Predicted probabilities were calculated holding the independent variables at their median.
